# HPV16 E7-Associated SERPINB3 Suppression and MYC-Related Epithelial Plasticity in Head and Neck Squamous Cell Carcinoma

**DOI:** 10.3390/cancers18152420

**Published:** 2026-07-27

**Authors:** Zengchen Liu, Siwei Zhang, Tianyang Liu, Yanjing Li, Rui Li, Huan Liu, Dongcun Wang, Yunyan Tang, Heng Ma, Yuting Zhang, Lanlan Wei, Ming Chu

**Affiliations:** 1 Department of Neurosurgery, Shenzhen Third People’s Hospital, The Second Affiliated Hospital, School of Medicine, Southern University of Science and Technology, Shenzhen 518112, China; 12233017@mail.sustech.edu.cn (Z.L.); eugenice@163.com (R.L.); 2 Open Shared Laboratory, National Clinical Research Center for Infectious Diseases, Shenzhen Third People’s Hospital, Shenzhen 518112, China; sw_zhang2528@163.com (S.Z.); liutianyang826@hotmail.com (T.L.); lyj0214056x@163.com (Y.L.); liuh120400@163.com (H.L.); tangyunyan1998@163.com (Y.T.); 18054336260@163.com (Y.Z.); 3 Laboratory of Medical Genetics, Harbin Medical University, Harbin 150081, China; 4 Department of Endodontics, The First Affiliated Hospital of Harbin Medical University, School of Stomatology, Harbin Medical University, Harbin 150001, China; 5 Department of Pathology, National Cancer Center/National Clinical Research Center for Cancer/Cancer Hospital & Shenzhen Hospital, Chinese Academy of Medical Sciences and Peking Union Medical College, Shenzhen 518116, China; wdctop@163.com; 6 Department of Head and Neck Surgery, National Cancer Center/National Clinical Research Center for Cancer/Cancer Hospital & Shenzhen Hospital, Chinese Academy of Medical Sciences and Peking Union Medical College, Shenzhen 518116, China; 07301020033@fudan.edu.cn

**Keywords:** head and neck squamous cell carcinoma, human papillomavirus, lymph node metastasis

## Abstract

**Background:** Human papillomavirus (HPV) infection defines a distinct subtype of head and neck squamous cell carcinoma (HNSCC). Although HPV-positive (HPV+) HNSCC generally shows better overall survival than HPV-negative (HPV−) disease, it is frequently associated with cervical lymph node metastasis. However, the epithelial cell states and viral gene-associated mechanisms underlying HPV−related metastatic progression remain incompletely understood. This study aimed to identify metastasis-associated epithelial subpopulations in HPV+ HNSCC and explore the potential role of HPV16 E7 in regulating metastatic programs. **Methods:** Public single-cell RNA-sequencing datasets from paired primary and metastatic HNSCC samples were integrated and analyzed to characterize malignant epithelial subpopulations. Copy number variation (CNV) inference, clustering, pathway enrichment, stemness scoring, and trajectory analysis were performed to define metastasis-associated epithelial states. TCGA transcriptomic data and tissue-based validation were used to support candidate gene screening. In vitro functional assays were performed using SERPINB3-knockdown and HPV16 early gene-overexpressing CAL27 cell models. **Results:** Single-cell analysis identified stem-like metastatic epithelial subpopulations in HPV+ and HPV− HNSCC. In HPV+ metastatic lesions, an ALDH2+/LAMB3+ epithelial subpopulation showed elevated epithelial–mesenchymal transition activity and stem-like features. SERPINB3 displayed a dynamic expression pattern during HPV+ metastatic progression. Functional assays showed that SERPINB3 knockdown enhanced CAL27 cell migration and invasion and was associated with activation of MYC- and epithelial–mesenchymal transition-related transcriptional programs. Among HPV16 early genes, E5, E6, E6*, and E7 showed different degrees of SERPINB3 suppression, while E7-expressing cells exhibited distinct transcriptional alterations associated with epithelial plasticity and metastatic-related programs. **Conclusions:** This study identifies a stem-like metastatic epithelial state in HPV-associated HNSCC and suggests a potential HPV16 early gene-SERPINB3-MYC-related regulatory mechanism involved in metastatic epithelial plasticity.

## Simple Summary

Human papillomavirus-positive head and neck cancer often has a better overall outcome than virus-negative disease, but many patients still develop lymph node metastasis, which complicates treatment and affects prognosis. The biological reasons why virus-positive tumors spread to lymph nodes remain unclear. In this study, we analyzed public single-cell and transcriptomic datasets and performed laboratory experiments to explore how human papillomavirus may influence metastatic behavior in head and neck cancer cells. We identified a stem-like tumor cell population associated with lymph node metastasis and found that HPV16 early genes may reduce SERPINB3 expression, with E7-associated changes linked to activation of MYC-related pathways and enhanced tumor cell migration.

## 1. Introduction

Cervical lymph node metastasis is common in HNSCC and represents a major adverse prognostic factor, critically influencing tumor staging, treatment selection, and patient survival [1,2]. Although epithelial–mesenchymal plasticity, lymphangiogenesis, and tumor-microenvironment interactions have been implicated in HNSCC lymph node metastasis, the epithelial cell-intrinsic programs that initiate metastatic dissemination remain incompletely defined [3,4,5,6]. Recent single-cell studies have identified rare epithelial subpopulations enriched for EMT and metastasis-associated signatures in HNSCC [3,4]. However, how these metastatic epithelial states are regulated in HPV-associated HNSCC, and whether HPV early genes directly reprogram these states to promote lymph node metastasis, remains unclear.

HPV-associated HNSCC, particularly oropharyngeal squamous cell carcinoma, represents a biologically distinct subgroup. Although HPV-associated tumors generally show favorable outcomes, cervical lymph node involvement remains common in a subset of patients [7,8]. HPV early genes, particularly E6 and E7, have been reported to regulate malignant transformation, EMT, immune responses, and metastatic behavior [9,10,11]. However, how HPV reshapes epithelial tumor cell states to promote lymph node metastasis remains poorly defined.

In this study, we integrated GEO single-cell datasets, TCGA transcriptomic data, and experimental validation to investigate HPV-associated lymph node metastasis in HNSCC. We identified an HPV+ tumor-enriched stem-like metastatic epithelial cluster and found SERPINB3 to be a key HPV-regulated metastasis-associated gene. Our findings further suggest that HPV16 E7-associated SERPINB3 suppression may contribute to MYC pathway activation and metastatic epithelial reprogramming, providing a potential regulatory axis underlying HPV-associated lymph node metastasis.

## 2. Materials and Methods

### 2.1. Patient Characteristics

A total of six sets of paired formalin-fixed paraffin-embedded (FFPE) tissue sections derived from HNSCC patients, each comprising a primary HNSCC specimen and the corresponding lymph node metastasis from the same patient, were collected from the Department of Head and Neck Surgery at the Cancer Hospital, Chinese Academy of Medical Sciences (Shenzhen Hospital), including three pairs from p16-negative (p16−) cases and three from p16-positive (p16+) cases. HPV-associated status in the clinical cohort was defined using p16 immunohistochemical positivity, a clinically accepted surrogate marker for HPV-associated HNSCC. p16 testing was performed in the accredited institutional clinical pathology laboratory. Direct HPV genotyping and HPV subtype information were not available for all clinical cases.

No participant data attrition occurred. Clinical data for HNSCC patients were collected from those enrolled at the Department of Head and Neck Surgery, Cancer Hospital of the Chinese Academy of Medical Sciences (Shenzhen Hospital). These data were merged with TCGA and UCSC database data for matching variables: age, sex, tumor site, smoking history, alcohol use, and lymph node metastasis status, for subsequent statistical analyses (Supplementary Table S1).

This study was approved by the Institutional Review Board (IRB) of Shenzhen Third People’s Hospital (approval number: 2021-055). All patient identifiers were anonymized, and personal information was strictly protected in accordance with the Declaration of Helsinki as revised in Fortaleza, Brazil, in October 2013.

### 2.2. scRNA Data Collection and Basic Analysis

scRNA-seq datasets from 18 cases were obtained from the Gene Expression Omnibus (GEO) database (GSE173468, GSE188737, GSE182227). Paired primary tumors (PTs) and metastatic tumors (MTs) sequencing data were selected for further integrated analysis. For quality control, cells with fewer than 200 features and genes expressed in three or fewer cells were removed. The scDblFinder (v1.20.0) package was used to remove doublets. All tumor data were split and quality-controlled based on feature counts and mitochondrial gene ratios. The R package harmony (v1.2.1) was used to integrate expression data from different samples, and Seurat (v5.1.0) was used for basic downstream analysis and visualization.

Specifically, SCTransform was used to normalize the quality-controlled data. Two thousand highly variable genes were identified and used for principal component analysis (PCA) to project cells into a low-dimensional space. The elbow plot was used to determine the number of principal components (PCs) to retain. The ‘RunHarmony’ function was then used to iteratively correct the low-dimensional PC representation of cells, setting the sample as a batch factor to reduce batch effects. The corrected PC matrix was used for unsupervised shared nearest neighbor-based clustering and UMAP visualization. The ‘FindAllMarkers’ function was used to identify marker genes for each subpopulation, and these were ranked based on the difference in the proportion of cells expressing the gene (pct.1–pct.2) [12].

Subclustering of major cell types was performed by subsetting the data for cells of interest and repeating the above process. Clusters were manually annotated based on marker genes, and contaminating cells were removed.

For comparison of the similarity between PT and MT-derived tumor cells, the PTs Seurat data were converted to a SingleCellExperiment file, and the normalized expression matrix was extracted as a reference. The ‘scmap’ (v1.28.0) package was used to compare differences between PTs and MTs, and the ‘getSankey’ function was used to visualize the results.

### 2.3. Copy Number Variation Analysis

CNV profiles were inferred using inferCNV v1.14.2, with T cells used as normal reference cells [13]. A CNV score was calculated for each epithelial cell based on the sum of squared scaled CNV signals. To retain the majority of tumor epithelial cells for downstream re-clustering, cells with CNV scores above the median minus one standard deviation were classified as malignant cells.

### 2.4. Pathway Enrichment Analysis

For malignant epithelial cells in the single-cell sequencing data, the top 200 genes from each subpopulation were selected, and the Metascape (v2023.12) database was used for functional enrichment analysis. The top 20 most significant pathways were visualized. The hallmark EMT pathway from the Molecular Signatures Database (MSigDB, v2023.2.Hs) was analyzed using the ‘GSVA’ package (v2.0.1) to obtain GSVA scores, which were visualized using ‘ggplot2’ and ‘ggboxplot’.

### 2.5. Cell Trajectory and CYTOTRACE2 Analysis

The ‘Monocle’ (v2.34.0) package was used to perform cell trajectory analysis based on differentially expressed genes in each cluster. The ‘CytoTRACE2’ (v1.0.0) package was used to analyze the differentiation potential of input cells [14]. The CytoTRACE2 scores were exported and visualized using ‘ggplot2’ (v3.5.1).

### 2.6. RNA-Seq Differential Analysis

The ‘DESeq2’ (v1.46.0) package was used to perform differential analysis on TCGA data and RNA-seq data involved in this study. Genes with a *p*-value < 0.05 and |Log2FC| > 1 were defined as differentially expressed genes (DEGs). Heatmaps and volcano plots of DEGs were visualized using the ‘ggplot’ and ‘pheatmap’ packages.

### 2.7. C1 Signature and shSERPINB3 Transcriptomic Overlap Analysis

To evaluate the transcriptional similarity between SERPINB3 knockdown and the HPV+C1 metastatic epithelial state, C1-representative genes were ranked according to the difference in the proportion of expressing cells between C1 and the other malignant epithelial clusters (pct.1–pct.2). The top 100 ranked genes were defined as the C1 signature. Of these, 96 genes were detected in the shSERPINB3 RNA-seq dataset and were retained for subsequent analysis. Upregulated genes following SERPINB3 knockdown were defined as genes with log2 fold change >1 and *p* < 0.05. Gene-set overlap and enrichment were evaluated using Fisher’s exact test, with genes detected in the RNA-seq dataset as the background gene set. Odds ratios and *p*-values were calculated, and the overlapping genes are provided in Supplementary Table S3.

### 2.8. Patient Subgrouping Based on Subpopulation Feature Gene Expression

The top 200 differentially expressed genes from the C1 cluster were extracted as a reference gene set, and the EPIC package (v1.1.7) was used to analyze subpopulation proportions in UCSC (GDC TCGA Head and Neck Cancer, HNSC) and GEO (GSE67614) data. Patients were grouped based on the median proportion of C1 in HPV+ MT’s epithelial cells. The ‘GSEA’ tool was used to score the EMT process for each patient, which was used to compare the EMT process and metastatic tendency between different C1 subgroups.

### 2.9. Cell Culture and Stable Cell Line Construction

CAL27 cells were cultured in DMEM supplemented with 10% FBS and maintained at 37 °C in a humidified incubator with 5% CO_2_.

For SERPINB3 knockdown, a short hairpin RNA (shRNA) targeting human SERPINB3 or a non-targeting negative control sequence was cloned into the lentiviral shRNA vector PGMLV-HU6-MCS-CMV-ZsGreen1-PGK-Puro (PGMLV-ZsGreen1-Puro). The target sequences used for the construction of the negative-control and SERPINB3-knockdown vectors are listed in Table 1. Untreated CAL27 cells were included as the blank control (BC), whereas CAL27 cells transduced with a non-targeting shRNA lentiviral vector served as the negative control (NC).

Recombinant lentiviral particles were produced in HEK-293T cells. CAL27 cells were infected with lentiviruses carrying either shSERPINB3 or the negative control shRNA, and transduction efficiency was monitored by ZsGreen1 fluorescence. Stably transduced cells were selected using puromycin to establish stable SERPINB3-knockdown and control cell lines. The knockdown efficiency of SERPINB3 was confirmed by qPCR and Western blotting.

For HPV16 gene overexpression, the cDNA sequences of HPV16 E6, HPV16 E6*, HPV16 E7, and HPV16 E5 were synthesized and individually cloned into the pLV-puro lentiviral expression vector (Supplementary Table S4). Recombinant lentiviruses carrying each HPV16 gene or the negative control vector were used to infect CAL27 cells, followed by puromycin selection to obtain stable polyclonal cell lines. For each HPV16 early gene, multiple biological replicates were independently established using the same corresponding lentiviral construct. Each replicate underwent independent cell seeding, lentiviral transduction, puromycin selection, and expansion. Stable cell lines were evaluated by cell growth and qPCR, and one line showing stable growth and robust target-gene expression was selected for subsequent RNA-seq analysis. 

### 2.10. RNA Extraction and RT-PCR

Total RNA was extracted using the HiPure Total RNA Midi Kit (Magen, Guangzhou, China) according to the manufacturer’s instructions. The RNA was reverse transcribed to cDNA using the Reverse Transcription Kit (TaKaRa, Kusatsu, Japan). A 20 μL reaction volume was prepared by mixing 1 μL of cDNA with TB Green Premix Ex Taq II (TaKaRa, Japan). qPCR was performed in a ViiA™ 7 instrument with the following conditions: 94 °C for 2 min, followed by 40 cycles of 94 °C for 20 s, 58 °C for 20 s, and 72 °C for 20 s. GAPDH or β-actin was used as an internal reference gene as appropriate for the corresponding qPCR assays. The primer sequences used for RT-qPCR are listed in Table 2.

### 2.11. Western Blot

For protein extraction, cells were washed twice with pre-chilled PBS, harvested by scraping, and lysed on ice using RIPA lysis buffer (Solarbio, Beijing, China) supplemented with PMSF. After centrifugation, the supernatant was collected, and protein concentration was measured using the BCA kit (Pierce, Rockford, IL, USA). For protein blotting, cell lysates were separated by SDS-PAGE and transferred to a PVDF membrane (IPVH00010, Millipore, Burlington, MA, USA). The membrane was blocked with 5% skim milk in Tris-buffered saline containing 0.1% Tween-20 (TBST) for 1.5 h at room temperature. The blot was then incubated with primary antibodies overnight at 4 °C, washed in TBST, and incubated with secondary antibodies for 2 h. Western blot analysis was performed using the statistical grayscale values of the blot.

### 2.12. Cell Scratch and Transwell Assays

Cells were seeded at a density of 1 × 10^5^ cells/mL in 6-well plates and allowed to grow until 95% confluence. Marks were made on the bottom of the plate, and scratches were made vertically with a 200 μL pipette tip from top to bottom. The cells were then washed with PBS. The first set of images was captured under a microscope at 0 h, and subsequent images were taken at the same location every 12 h. Each group of cells was subjected to three replicate experiments, and statistical differences were analyzed using GraphPad Prism (version 9.0.0).

Transwell invasion assays were conducted to determine the effect of SERPINB3 knockdown on the invasive capacity of CAL27 cells. Cells were seeded in 24-well Transwell inserts (Corning, Corning, NY, USA) coated with a pre-laid matrix gel and containing serum-free medium. The lower chamber was filled with medium containing FBS. After 24 h, cells that did not migrate through the membrane were removed, and the cells that had migrated through the membrane were fixed with formaldehyde and stained with crystal violet. Once the membrane was dry, it was removed and mounted with a 5% resin. The membranes were then examined and photographed under a microscope, and the number of cells on the membrane was counted using ImageJ. Statistical differences were analyzed using GraphPad Prism (version 9.0.0).

### 2.13. Immunohistochemistry (IHC) and In Situ Hybridization (ISH) Staining Evaluation

IHC was performed on formalin-fixed, paraffin-embedded HNSCC tissue sections. Following deparaffinization, rehydration, and antigen retrieval, sections were incubated overnight at 4 °C with primary antibodies against SERPINB3 (Proteintech, Wuhan, China, 26558-1-AP, 1:1000). HRP-conjugated secondary antibodies were applied (Proteintech, Wuhan, China, RGAM601), and slides were scanned using the Vectra Automated Quantitative Pathology System (PerkinElmer, Shelton, CT, USA).

After staining, the six paired specimens, comprising three p16+ and three p16− cases with matched primary tumor and lymph node metastatic tissues, were independently evaluated by two pathologists. During tumor-region identification and IHC assessment, both pathologists were blinded to the p16 status, primary or metastatic origin, and experimental grouping of the tissue sections. The results were analyzed using the IHC Profiler plugin in ImageJ software (v1.52), in which high-positive, positive, low-positive, and negative staining categories were assigned scores of 4, 3, 2, and 1, respectively [15]. The final IHC score was calculated according to the distribution of staining-intensity categories generated by the IHC Profiler. For RNA in situ hybridization (ISH), staining was evaluated according to the manufacturer’s guidelines, with positivity defined as at least one detectable signal per cell within the tumor region.

### 2.14. Statistical Analysis

All statistical analyses were performed using GraphPad Prism version 9.0.0 and R software version 4.4.2. For group comparisons, Student’s *t*-test or chi-square test was applied as appropriate based on data characteristics. Pearson correlation analysis was used for normally distributed variables, while Spearman rank correlation was employed for non-normally distributed data. Statistical significance was defined as a two-sided *p*-value less than 0.05. Univariable and multivariable logistic regression analyses were performed to evaluate the association between p16 status and lymph node metastasis. The multivariable model was adjusted for age, sex, anatomical subsite, smoking history, and alcohol consumption. Odds ratios (ORs), 95% confidence intervals, and two-sided *p*-values were reported. Significance levels were denoted as follows: * *p* < 0.05, ** *p* < 0.01, *** *p* < 0.001, and **** *p* < 0.0001.

## 3. Results

### 3.1. Clinical Characteristics of p16-Defined HPV-Associated HNSCC

We first integrated 81 HNSCC cases from our institutional cohort with 430 TCGA-HNSCC cases, yielding a total sample size of 511 patients. In accordance with the available clinical annotations, p16 positivity was used to define HPV-associated status. p16-defined HPV-associated status was identified in 20.4% (104/511) of cases, with the remaining cases classified as p16−. Demographic analysis (Table 3, Supplementary Table S1) revealed a male predominance (75.5%, 386/511) across the cohort, with 85.5% of patients aged ≥50 years. p16− patients showed a higher mean age than p16+ patients (61.24 ± 11.42 vs. 58.15 ± 9.39 years, *p* = 0.047).

Anatomic distribution analysis identified the oral cavity as the most frequent primary site in the overall cohort (276/511, 54.0%). Significant differences in tumor localization were observed between p16+ and p16-groups (*p* = 0.001). p16+ tumors showed a higher proportion of oropharyngeal tumors compared with p16− tumors (43.3% vs. 5.7%), whereas p16− tumors were more frequently located in the oral cavity (60.4% vs. 28.8%) and larynx (31.4% vs. 22.1%).

Chi-square analysis showed that p16+ tumors had a higher proportion of lymph node metastasis than p16− tumors (65.4% vs. 54.1%, *p* = 0.046). Consistently, univariate logistic regression indicated an association between p16+ status and lymph node metastasis. However, after adjustment for age, sex, anatomical subsite, smoking history, and alcohol consumption, this association did not remain statistically significant in our cohort (adjusted OR = 1.49, *p* = 0.108; Supplementary Table S2). These findings suggest a potential association between p16+ status and lymph node involvement, while the conclusion that HPV-associated status independently promotes lymph node metastasis requires further validation in larger, anatomically stratified cohorts.

### 3.2. Single-Cell Characterization of Primary and Metastatic HNSCC

**Figure 1:**
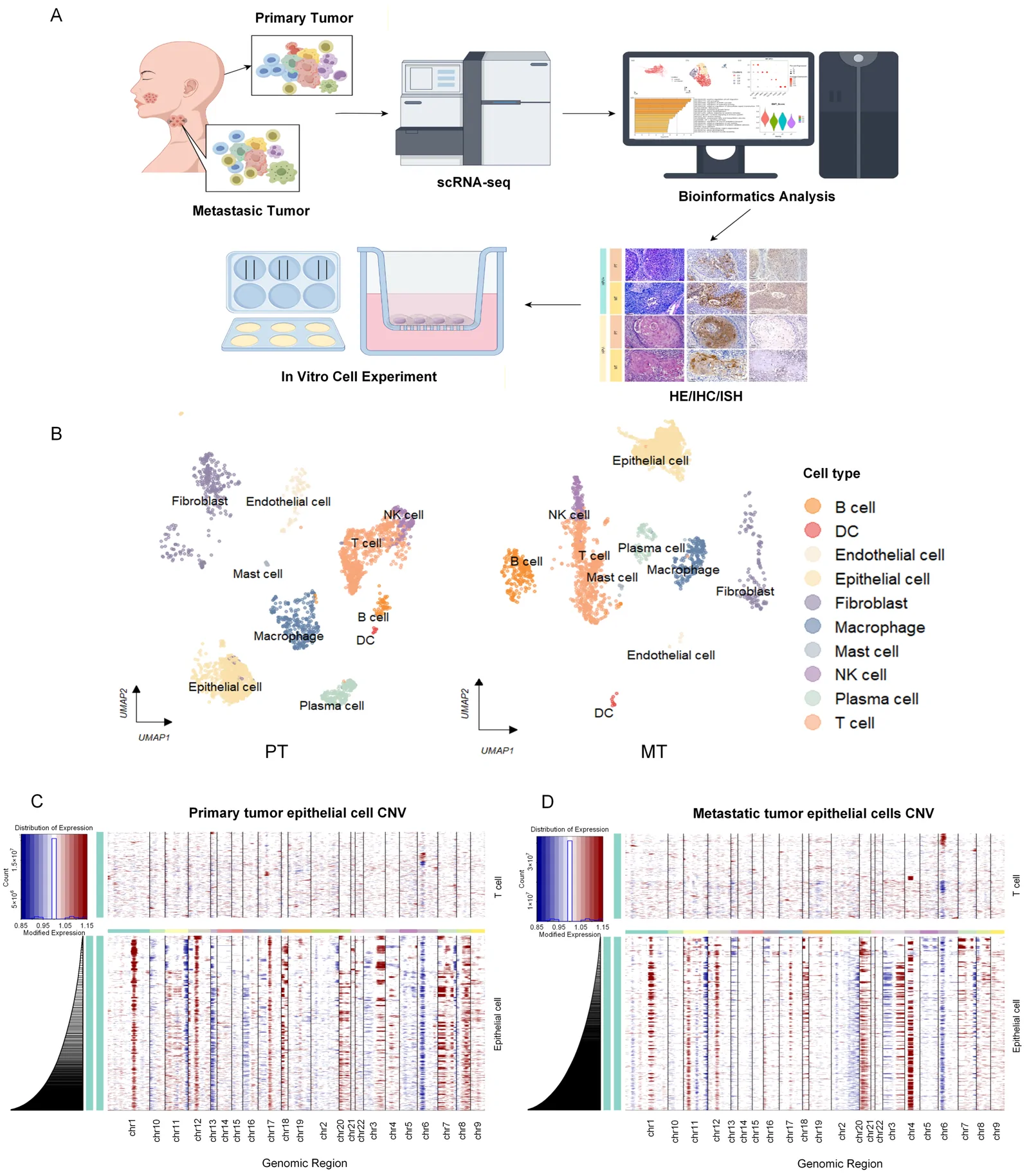
Comprehensive cellular overview of primary and metastatic HNSCC. (**A**) Schematic diagram of the study process; (**B**) UMAP plots of cell type annotations for PTs (primary tumors) and MTs (metastatic tumors); (**C**,**D**) Chromosomal copy number variation (CNV) heatmaps of epithelial cells in PT and MT samples.

To investigate HPV-associated epithelial programs related to lymph node metastasis, we integrated and analyzed single-cell sequencing data from three GEO datasets, encompassing sequencing data from 2 HPV+ and 7 HPV− HNSCC cases. After performing quality control and batch effect removal on all cells, we merged the three datasets, resulting in a total of 60,375 cells (Figure 1A–D). We primarily identified 10 distinct cell types, including: B-cells (MS4A1, CD79A), dendritic cells (CLEC4C, LILRA4), endothelial cells (ENG, VWF), epithelial cells (KRT19, KRT5), fibroblasts (COL1A1, MYL9), macrophages (CD68, CD14), mast cells (TPSAB1, KIT), NK cells (KLRD1, IL2RB), plasma cells (IGHG1, MZB1), and T-cells (CD3E, PTPRC) (Figure S1C).

Based on the collected single-cell data, epithelial cells consistently constituted the predominant component of tumor tissues in both PTs and MTs, with an even higher proportion observed in HPV+ HNSCC, followed by T cells (Figure S1A,B). Given the dominant presence of epithelial cells and their established role in driving tumor metastasis, subsequent analyses were focused on epithelial cell states and functional differences.

CNV analysis of epithelial cells isolated from PT and MT sites revealed largely conserved genomic profiles, despite localized disparities on chromosomes 2 and 4 (Figure 1C,D). This genomic consistency supports the subsequent focus on MT-derived epithelial cells to investigate key drivers of metastasis.

### 3.3. ALDH2+/LAMB3+ Epithelial Cluster Shows Stem-like and EMT-Associated Features in HPV+ Metastases

**Figure 2:**
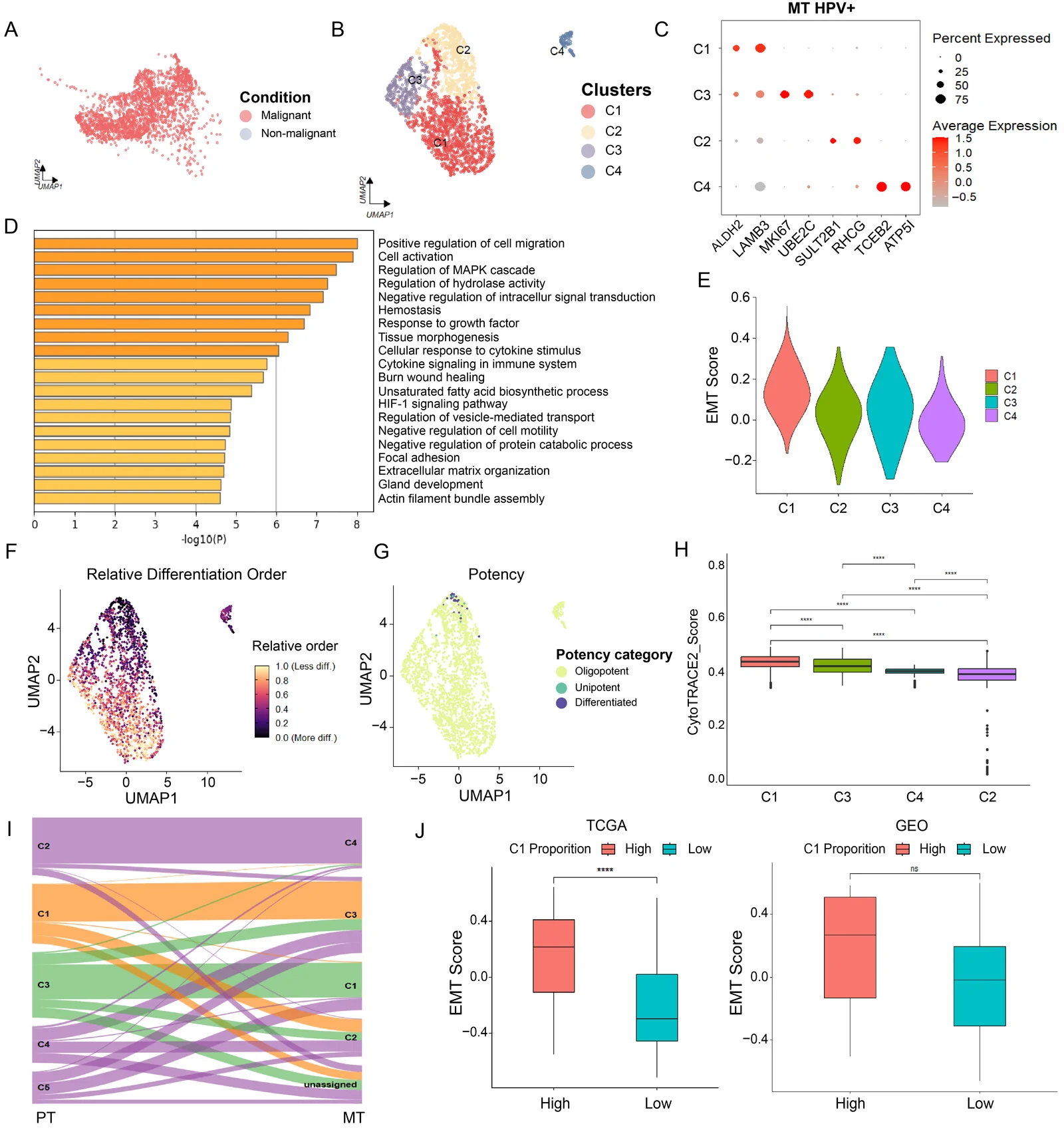
Identification of lymph node metastasis subgroups in HPV+ HNSCC. (**A**) UMAP plot distinguishing malignant from non-malignant epithelial cells in MTs; (**B**) UMAP plot of the reclustering of malignant epithelial cells in MTs; (**C**) Markers of the reclustered malignant epithelial subclusters in MTs; (**D**) Functional enrichment results of specific genes in the C1 subgroup; (**E**) Violin plot of EMT scores among malignant epithelial subgroups; (**F**) UMAP visualization of CytoTRACE2 relative differentiation order in HPV+ metastatic malignant epithelial cells. (**G**) UMAP visualization of CytoTRACE2-predicted potency categories. (**H**) Boxplot comparing CytoTRACE2 scores across malignant epithelial subgroups; (**I**) Sankey diagram showing the correspondence between HPV+ HNSCC PTs and MTs subpopulations, with each line representing a single cell; “unassigned” refers to the subpopulation in MT with no corresponding relationship; (**J**) Boxplots of EMT scores in TCGA and GEO datasets stratified by the median proportion of C1 subpopulation in patient-derived epithelial cells; Wilcoxon test was used for significance analysis; ****, *p* < 0.0001.

Based on CNV scoring, all HPV+ HNSCC epithelial cells were categorized into malignant and normal epithelial cells. Upon re-clustering the malignant tumor cells, four distinct clusters were identified (Figure 2A–C). Functional enrichment analysis of these clusters revealed that the differential genes in the C1 cluster (ALDH2+/LAMB3+) were primarily enriched in pathways related to positive regulation of cell migration and cell adhesion (Figure 2D). Given that epithelial tumor metastasis is often mediated by the EMT process, the EMT activity of the four subpopulations was scored (Figure 2E). It was found that, compared to other subpopulations, C1 exhibited a more significant EMT gene expression profile.

A number of reports have indicated that cancer stem-like cells are closely associated with tumor metastasis [16,17,18,19]. CytoTRACE2 analysis was performed to estimate the differentiation potential of malignant epithelial cells in metastatic tumors. According to the CytoTRACE2-predicted potency categories, a large proportion of malignant epithelial cells in metastatic tumors were classified as oligopotent-like, with the C1 cluster showing relatively higher CytoTRACE2 scores than the other clusters (Figure 2F,H). Additionally, to confirm that the C1 cluster originated from PTs, the similarities between different clusters in PTs and MT were analyzed. It was found that the C3 cluster in PTs shared more similar features with the C1 cluster in MTs, indicating the origin of the C1 cluster. Together, these findings suggest that the ALDH2+/LAMB3+ C1 cluster represents a metastasis-associated epithelial subpopulation with EMT-associated and CytoTRACE2-predicted stem-like features in HPV+ HNSCC.

To verify the universality of the metastatic cluster’s characteristics, we collected RNA-seq data from HNSCC in the TCGA and GEO databases for validation. Patients were categorized based on the features of the C1 cluster into high-C1 and low-C1 groups, and EMT scores were evaluated for each group. Analysis of TCGA data showed that patients with a high C1 proportion had higher EMT scores, indicating greater metastatic propensity (Figure 2J), while GEO data, though showing no significant intergroup differences, revealed a consistent trend of higher EMT scores in high-C1 patients (Figure 2J).

### 3.4. Dynamic SERPINB3 Expression During HPV-Associated Metastatic Progression

**Figure 3:**
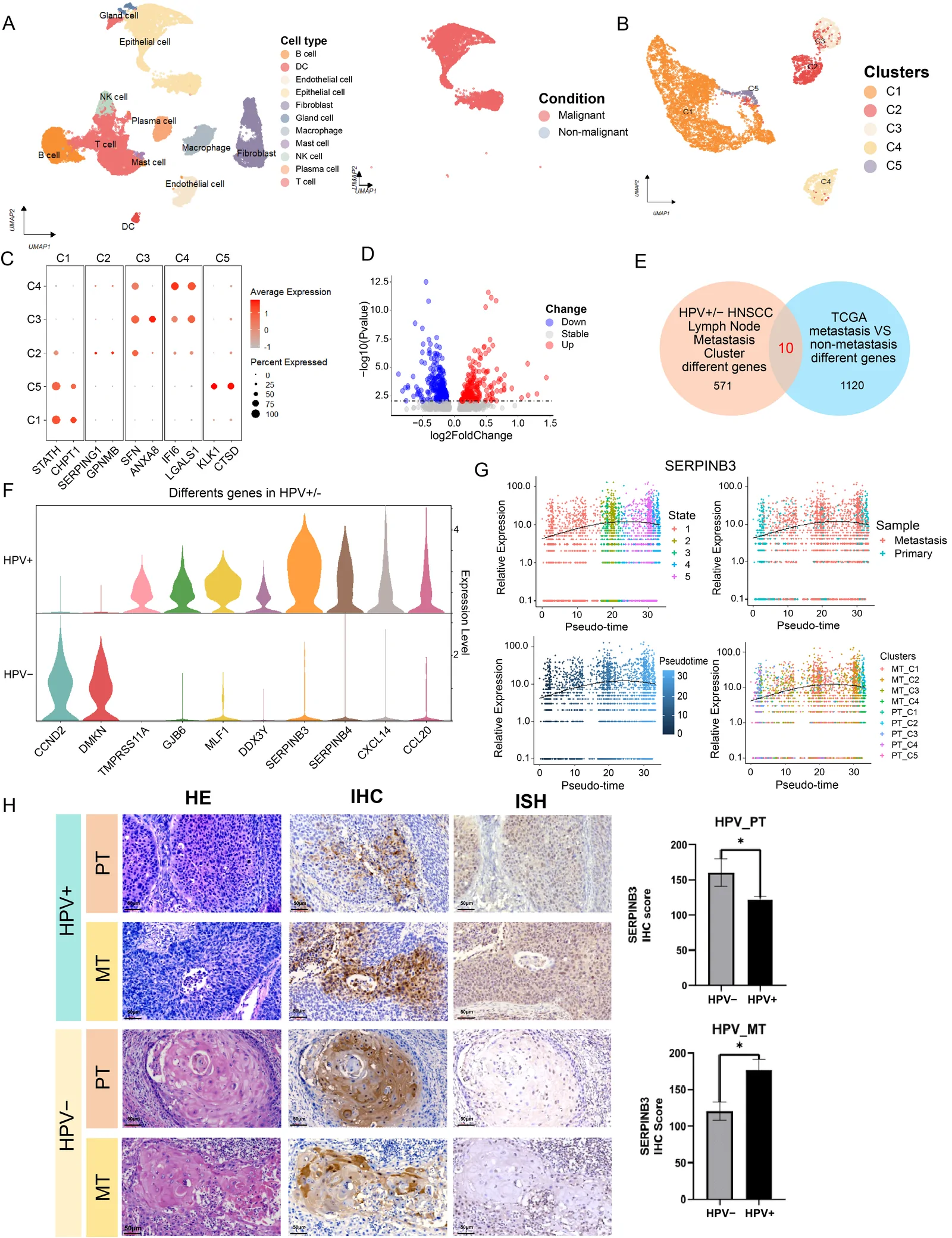
Analysis of HPV-related regulatory gene screening and expression of SERPINB3. (**A**) UMAP visualization of annotated HPV− HNSCC cell populations. The right panel shows copy number variation (CNV)-based classification of epithelial cells into malignant and non-malignant populations; (**B**) Reclustering of HPV− MT malignant epithelial cells; (**C**), Representative gene markers of HPV− MT malignant epithelial cell subgroups; (**D**) Volcano plot of differential analysis according to lymph node metastasis in TCGA; (**E**) Venn diagram of differential genes in HPV+ and HPV− HNSCC metastatic subgroups and TCGA lymph node metastasis-related genes; (**F**) Violin plot of expression levels of 10 differential genes in HPV+ MT epithelium; (**G**) Curve chart of SERPINB3 expression variation with pseudotime; (**H**) SERPINB3 expression in HPV+ and HPV− HNSCC PT/MT sections as shown by HE, IHC, and ISH staining, and statistical differences using ImageJ; *, *p* < 0.05.

To compare metastasis-associated epithelial programs between HPV+ and HPV− HNSCC, we analyzed CNV-defined malignant epithelial cells from HPV− metastatic tumors using a similar computational workflow (Figure 3A–C; Figure S2A–F). Reclustering analysis identified the C3 cluster, characterized by SFN and ANXA8 expression, as a metastasis-associated epithelial subpopulation in HPV− tumors. Functional enrichment analysis showed that C3 marker genes were enriched in EMT-related pathways and VEGFR signaling (Figure S2A,B). In addition, CytoTRACE2 analysis indicated that the HPV− C3 subpopulation showed relatively higher differentiation potential and was classified within the CytoTRACE2-predicted oligopotent-like category compared with other HPV− metastatic epithelial subgroups (Figure S2C–E). Subsequent intersectional analysis of differentially expressed genes between the HPV+ C1 cluster and the HPV− C3 cluster revealed a set of HPV-associated metastasis-related candidate genes.

To identify genes driving lymph node metastasis in HNSCC, we conducted two parallel differential expression analyses. First, we compared the transcriptomic profiles of HPV+ and HPV− HNSCC lymph node metastasis subpopulations, resulting in 571 differentially expressed genes associated with HPV status. Second, we analyzed TCGA HNSCC cohort transcriptomes stratified by lymph node metastasis status, identifying 1120 genes differentially expressed between metastatic and non-metastatic tumors (Figure 3D). Venn diagram analysis of the overlapping genes from these two comparisons revealed 10 core HPV-regulated metastasis driver genes (Figure 3E). Among these, CCND2 and DMKN exhibited preferential overexpression in HPV− MT, whereas SERPINB3/B4 dominated HPV+ MT contexts (Figure 3F).

Based on the findings reported by Huang et al., in which HPV was shown to enhance chemoresistance in HNSCC by suppressing SERPINB3 expression, we hypothesized that HPV may also promote early metastatic dissemination by downregulating SERPINB3 [20]. To test this hypothesis, we performed pseudotime trajectory analysis of malignant cells from PT and MT. SERPINB3 exhibited a biphasic expression pattern along the metastatic trajectory, with reduced expression during the early metastatic transition but marked upregulation in established metastatic lesions (Figure 3G). Consistently, IHC analysis showed that SERPINB3/B4 expression was significantly increased in HPV+ MT compared with matched PT, whereas this pattern was not observed in HPV− metastatic lesions (Figure 3H).

To further validate the transcriptional regulatory effect of HPV16 early oncogenes on downstream gene expression in CAL27 cells, we separately overexpressed HPV16 E5, E6, E6*, and E7 in CAL27 cells and performed transcriptome sequencing. Functional enrichment analysis of differentially expressed genes was implemented via the Metascape tool, and the top 20 enriched GO biological process terms and KEGG pathways for upregulated and downregulated genes in each overexpression group are presented in Figure S3.

### 3.5. SERPINB3 Knockdown Enhances CAL27 Cell Migration and Invasion

**Figure 4:**
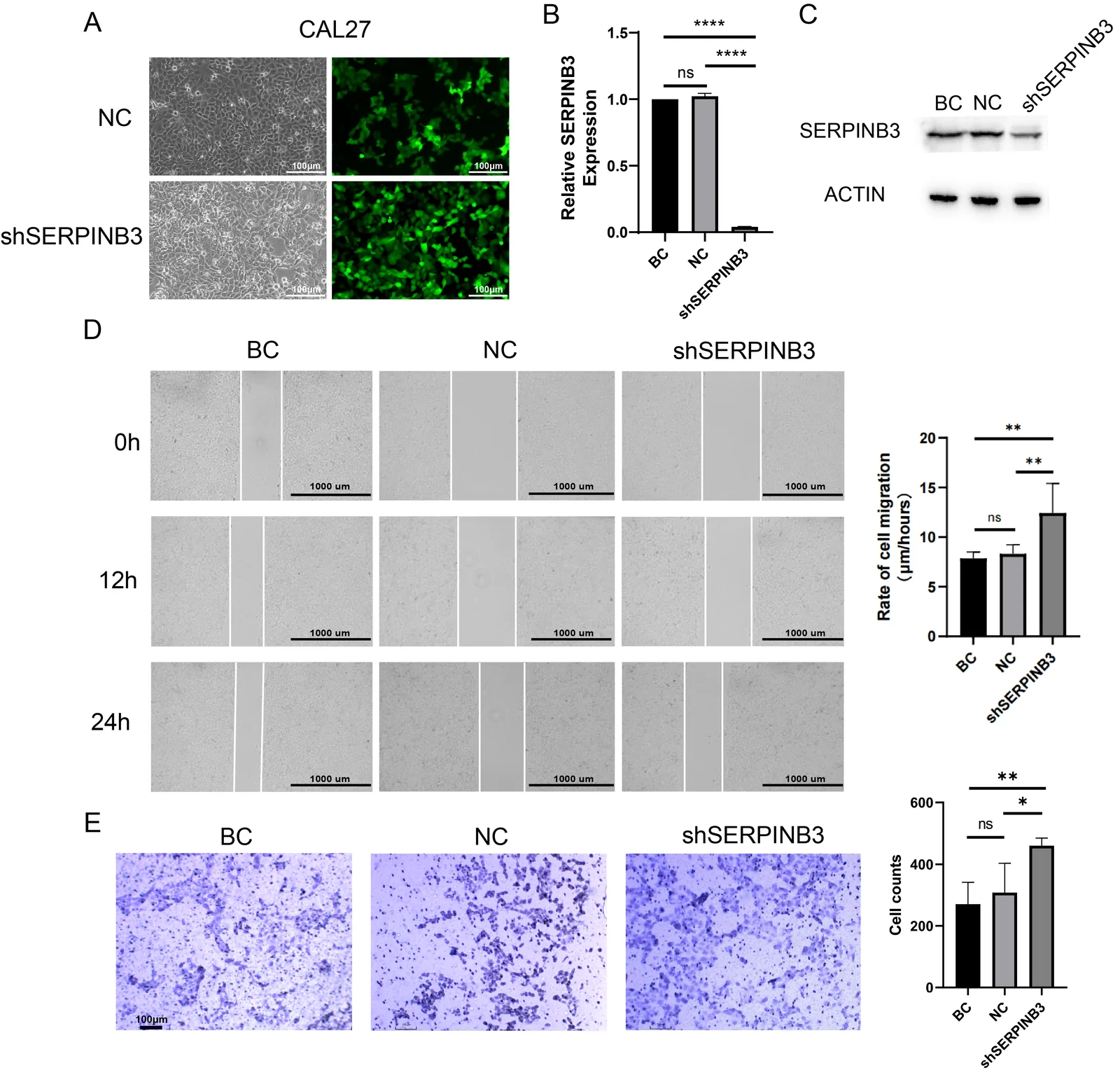
SERPINB3 knockdown enhances CAL27 cell migration and invasion. (**A**) Representative ZsGreen1 fluorescence images of CAL27 cells transduced with lentiviral shSERPINB3 or control vectors. (**B**,**C**) qPCR and Western blot validation of SERPINB3 knockdown efficiency in blank control (BC), negative control (NC), and shSERPINB3 CAL27 cells. (**D**) Representative wound-healing assay images and quantification showing enhanced lateral migration after SERPINB3 knockdown. (**E**) Representative images and quantification of matrix gel-coated Transwell invasion assays showing an increased number of cells invading through the membrane after SERPINB3 knockdown. **** *p* < 0.0001; ** *p* < 0.01; * *p* < 0.05.

To functionally investigate the role of SERPINB3 in HNSCC cell motility, we established stable SERPINB3-knockdown CAL27 cells using lentiviral shRNA transduction. Successful lentiviral infection was indicated by robust fluorescent reporter expression in CAL27 cells (Figure 4A). The knockdown efficiency of SERPINB3 was further confirmed at both the mRNA and protein levels by qPCR and Western blotting, respectively (Figure 4B,C).

Functional assays revealed that SERPINB3 depletion significantly enhanced the migratory capacity of CAL27 cells. In the wound-healing assay, SERPINB3-knockdown cells exhibited accelerated wound closure compared with both blank control and negative control cells, indicating increased lateral migration ability (Figure 4D). Consistently, matrigel-coated Transwell invasion assays showed a marked increase in the number of cells invading through the matrix-coated membrane upon SERPINB3 knockdown (Figure 4E). Together, these results suggest that loss of SERPINB3 promotes the migratory and invasive potential of CAL27 cells, supporting a suppressive role of SERPINB3 in HNSCC cell motility.

### 3.6. SERPINB3 Knockdown Activates MYC-Related Transcriptional Programs

**Figure 5:**
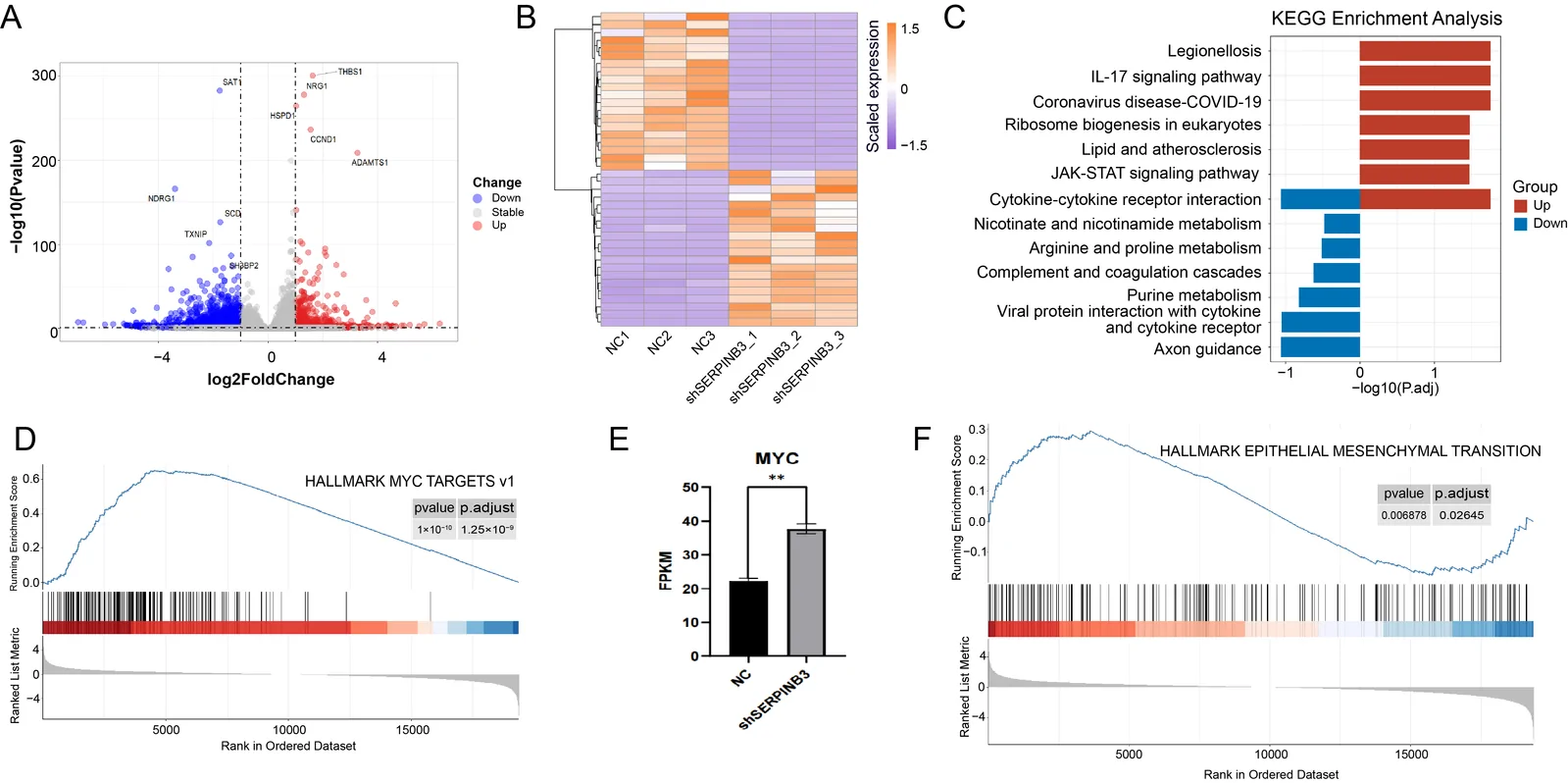
SERPINB3 knockdown activates MYC-related transcriptional programs. (**A**) Volcano plot showing differentially expressed genes between shSERPINB3 and negative-control (NC) CAL27 cells. Red, blue, and gray dots indicate upregulated, downregulated, and unchanged genes, respectively. (**B**) Heatmap of representative differentially expressed genes. Orange and purple indicate relatively high and low scaled expression, respectively. (**C**) KEGG pathway enrichment analysis of differentially expressed genes. Red and blue bars indicate pathways enriched among upregulated and downregulated genes, respectively. (**D**) GSEA enrichment plot showing activation of the MYC pathway in shSERPINB3 cells. (**E**) MYC expression based on RNA-seq data from shSERPINB3 and control cells. (**F**) GSEA enrichment plot showing activation of the EMT pathway in shSERPINB3 cells. In (**D**,**F**), the blue curves indicate the running enrichment scores, the black vertical lines mark the positions of gene-set members, and the red-to-blue gradients represent positive-to-negative ranked-list metrics. ** *p* < 0.01.

Transcriptomic profiling of shSERPINB3 and control CAL27 cells identified 605 upregulated and 1643 downregulated differentially expressed genes (DEGs) following SERPINB3 knockdown (Figure 5A,B). Functional enrichment analysis showed that the upregulated genes were enriched in IL-17 and JAK-STAT activation (Figure 5C). RNA-seq further confirmed increased MYC expression (Figure 5E). Consistently, GSEA revealed significant enrichment of MYC- and EMT-associated transcriptional programs in shSERPINB3 cells compared with control cells (Figure 5D,F). These results suggest that SERPINB3 depletion promotes MYC/EMT-related epithelial plasticity in CAL27 cells. A supplementary overlap analysis showed a trend toward overlap between shSERPINB3-upregulated genes and the HPV+ C1 signature, although this did not reach statistical significance (odds ratio = 2.16, *p* = 0.053; Supplementary Table S3).

### 3.7. HPV16 Early Gene-Mediated SERPINB3 Regulation and E7-Associated Metastatic Transcriptional Remodeling

**Figure 6:**
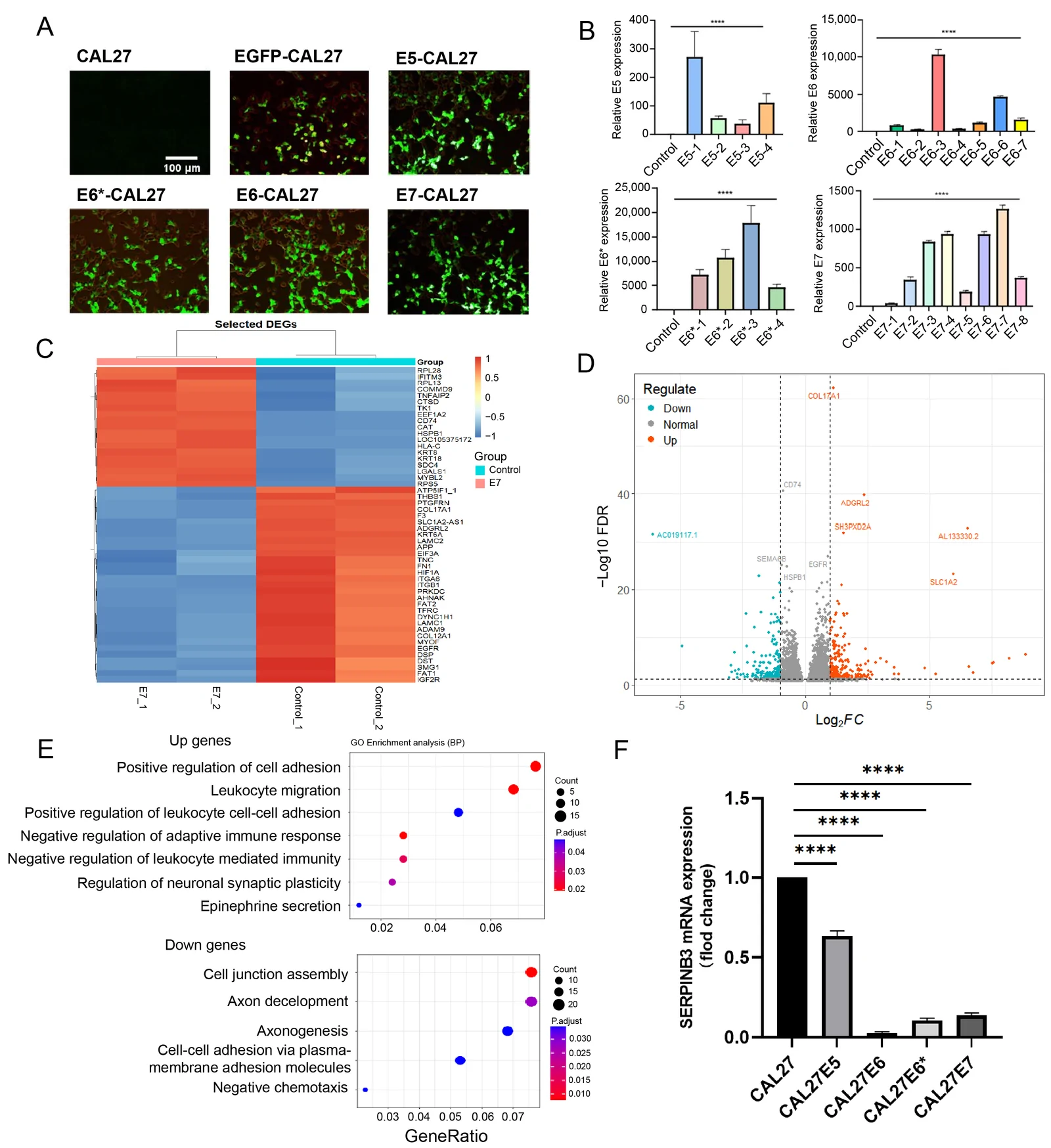
HPV16 early genes suppress SERPINB3 expression, with E7-associated pro-migratory transcriptional remodeling. (**A**) Representative fluorescence images showing transduction efficiency of HPV16 early gene-expressing CAL27 cell lines. (**B**) qPCR validation of HPV16 E5-, E6-, E6*-, and E7-expressing CAL27 stable cell lines. The numerical suffixes indicate independently established biological replicates generated using the same corresponding lentiviral construct; they do not represent different primer pairs or technical replicates. (**C**) Heatmap of differentially expressed genes between E7-expressing and control CAL27 cells. (**D**) Volcano plot showing differentially expressed genes in E7-expressing cells compared with controls. (**E**) GO enrichment analysis of upregulated and downregulated genes in E7-expressing cells. (**F**) qPCR analysis of SERPINB3 expression in control and HPV16 early gene-expressing CAL27 cells. ****, *p* < 0.0001.

To investigate whether HPV16 early genes influence SERPINB3 expression and metastatic-related transcriptional programs, we ectopically expressed HPV16 E5, E6, E6*, and E7 in the HPV− CAL27 cell line as a controlled gain-of-function model. Successful transduction was confirmed by fluorescence microscopy and qPCR detection of the corresponding viral genes (Figure 6A,B). Transcriptomic profiling showed that E7-expressing CAL27 cells exhibited a distinct expression pattern compared with control cells (Figure 6C,D). Functional enrichment analysis revealed that E7 upregulated pathways related to cell adhesion and leukocyte migration, while downregulated pathways were enriched in cell junction assembly and cell–cell adhesion, suggesting a shift toward a pro-migratory epithelial state (Figure 6E). qPCR validation demonstrated that HPV16 early gene expression resulted in reduced SERPINB3 levels to different extents, with E7 showing a functional association with metastatic-related transcriptional remodeling (Figure 6F). Together, these findings indicate that HPV16 early genes may promote metastatic epithelial reprogramming, at least in part, through SERPINB3 suppression.

## 4. Discussion

Integrated clinical analysis showed that the study cohort predominantly comprised male patients aged ≥50 years. In univariate analysis, p16+ tumors showed a higher proportion of lymph node metastasis than p16− tumors, consistent with previous reports [21]. However, this association was no longer statistically significant after adjustment for age, sex, anatomical subsite, smoking history, and alcohol consumption. Therefore, our findings support an unadjusted association between p16 positivity and lymph node involvement rather than an independent effect of HPV-associated status on metastasis. No significant differences in smoking or alcohol consumption were observed between the two groups; however, this does not establish that HPV-related carcinogenesis occurs independently of these conventional risk factors. Potential interactions among HPV status, tobacco exposure, alcohol consumption, and anatomical subsite require further investigation in larger, prospectively collected cohorts.

The persistent challenges of LNM, disease recurrence, and postoperative complications in HPV+ HNSCC continue to represent critical clinical hurdles requiring resolution in contemporary therapeutic management [22,23]. Our identification of HPV+ C1 and HPV− C3 as distinct metastasis-associated epithelial clusters suggests that HPV+ and HPV− HNSCC may involve different metastatic epithelial programs. The C1 subpopulation in HPV+ malignant epithelial cells showed elevated EMT pathway activity together with CytoTRACE2-predicted oligopotent-like features, suggesting a more plastic, metastasis-associated epithelial state characterized by EMT activation and reduced differentiation status. This interpretation is consistent with previous studies showing that epithelial–mesenchymal transition programs are closely linked to cancer stem-like states and tumor metastasis [24,25].

In HPV− HNSCC, the C3 metastasis-associated subpopulation also showed CytoTRACE2-predicted oligopotent-like features in the supplementary analysis, but its enriched pathways were distinct from those observed in HPV+ C1. In particular, C3 marker genes were enriched in EMT-related pathways and VEGFR signaling, suggesting that HPV− HNSCC may involve distinct metastasis-associated programs, potentially related to angiogenesis-associated remodeling and tumor cell dissemination. These findings support the presence of divergent metastatic epithelial states between HPV+ and HPV− HNSCC, while further functional studies are needed to determine whether these transcriptional programs directly contribute to metastatic dissemination [26,27].

SERPINB3, a member of the serine protease inhibitor family, primarily regulates keratinization processes in normal cells. However, its overexpression in squamous cell carcinoma has been frequently associated with tumorigenesis and progression [28]. Building upon previous findings, we identified two distinct regulatory roles of SERPINB3 in HNSCC pathogenesis. First, as demonstrated by Huang et al., elevated SERPINB3 expression in HPV− HNSCC upregulates the Fanconi anemia pathway to enhance DNA double-strand break repair, thereby sustaining cell survival and promoting chemoresistance [20]. Conversely, in HPV-associated HNSCC, HPV16 early gene-associated SERPINB3 suppression, particularly in E7-expressing cells, may contribute to EMT-related epithelial plasticity and metastatic potential. These differential regulatory mechanisms align with the characteristic clinical features of HPV− HNSCC (enhanced chemoresistance) [29] and HPV+ HNSCC (increased propensity for LNM), collectively underscoring possible contributions of SERPINB3 to metastatic progression during squamous cell carcinoma development.

Our findings further suggest that SERPINB3 suppression may contribute to MYC pathway activation during HPV-associated metastatic progression. In SERPINB3-knockdown CAL27 cells, transcriptomic analysis showed enrichment of MYC-related signaling, accompanied by enhanced migratory and invasive capacities. These observations are consistent with previous studies showing that MYC promotes HNSCC progression and metastasis, partly through regulation of EMT-associated programs such as SNAIL and vimentin [30,31]. In parallel, HPV16 E7 overexpression markedly reduced SERPINB3 expression and induced transcriptional changes associated with cell adhesion and junction remodeling, suggesting that E7 may facilitate a pro-metastatic epithelial state through SERPINB3 suppression. Notably, direct comparison with the HPV+ C1 signature suggested that SERPINB3 knockdown did not fully reproduce the complete patient-derived metastatic epithelial program, indicating that additional HPV-associated or microenvironmental factors may also be required.

Based on these findings, we propose that HPV16 E7-associated SERPINB3 suppression may contribute to MYC-related metastatic programs and epithelial plasticity. This model provides a potential mechanistic link between HPV early gene activity, SERPINB3 suppression, MYC pathway activation, and lymph node metastasis in HPV+ HNSCC. However, the precise molecular mechanism by which E7 represses SERPINB3, and whether MYC activation is directly required for SERPINB3 loss-induced metastasis, remain to be further investigated.

This study has several limitations. First, the number of HPV+ HNSCC cases with paired primary and metastatic lesions in public single-cell datasets was limited, and larger independent cohorts are needed for further validation. Second, CAL27 is an HPV− cell line; therefore, the HPV16 early gene-overexpression experiments should be interpreted as a controlled gain-of-function model rather than a complete representation of native HPV+ HNSCC. Preliminary evaluation of several HPV+ HNSCC cell lines revealed substantial heterogeneity in basal SERPINB3 expression, limiting direct comparisons across unrelated cellular backgrounds. Third, although our data suggest that HPV16 early genes, particularly E7, may suppress SERPINB3 expression and promote MYC and EMT-related epithelial plasticity, the molecular mechanism underlying SERPINB3 repression remains unresolved. In addition, this study focused on HPV16, and whether other high-risk HPV subtypes share similar regulatory mechanisms requires further investigation. Finally, the functional evidence was mainly derived from in vitro models and tissue-based validation, and in vivo studies are needed to further evaluate the role of the HPV16 early gene-SERPINB3-MYC axis in lymph node metastasis.

## 5. Conclusions

This work identified an ALDH2^+^/LAMB3^+^ stem-like epithelial subset featuring strong EMT activity as a key metastatic cell population within lymph node lesions of HPV+ HNSCC. Mechanistic exploration suggested that HPV16 E7-associated SERPINB3 suppression may contribute to MYC-related transcriptional activation and metastatic epithelial plasticity. These results provide a potential HPV E7-SERPINB3-MYC regulatory axis for understanding HPV-associated lymph node metastasis in HNSCC and may offer candidate biomarkers or therapeutic directions for future investigation.

## Figures and Tables

**Table 1:** Target sequences used for shRNA-mediated SERPINB3 knockdown.

Target	TargetSeq
NC	TTCTCCGAACGTGTCACGT
shSERPINB3	AGAGAGACACGTGTCGATTTA

**Table 2:** Primer sequences used for RT-qPCR.

Primer Name	Sequence
β-Actin-F	AGTGTGACGTGGACATCCGCAAAG
β-Actin-R	ATCCACATCTGCTGGAAGGTGGAC
SERPINB3-F	CGCGGTCTCGTGCTATCTG
SERPINB3-R	ATCCGAATCCTACTACAGCGG
E6*-F	GCAACAGTTACTGCGACGTG
E6*-R	CAACAAGACATACATCGACCG
E6-F	GAACAGCAATACAACAAACCG
E6-R	CCACCGACCCCTTATATTATG
E7-F	CAGCTCAGAGGAGGAGGATG
E7-R	CACAACCGAAGCGTAGAGTC
H_GAPDH-F	GTCTCCTCTGACTTCAACAGCG
H_GAPDH-R	ACCACCCTGTTGCTGTAGCCAA
E5-R	GCAGAGGCTGCTGTTATCCAC
E5-F	CCACAACATTACTGGCGTGC

**Table 3:** Clinical information statistics of HNSCC patients.

Clinical Characteristic	p16-Defined HPV-Associated Status		
	p16 Negative 407 (79.6%)	p16 Positive 104 (20.4%)	*p*-Value
Age (mean ± SD)	61.24 ± 11.42	58.15 ± 9.39	0.047
<40	11 (2.7%)	4 (3.8%)	
40 ≤ - < 50	46 (11.3%)	13 (12.5%)	
50 ≤ - < 60	121 (29.7%)	43 (41.3%)	
≥60	229 (56.3%)	44 (42.4%)	
Sex			0.015
Male	298 (73.2%)	88 (84.6%)	
Female	109 (26.8%)	16 (15.4%)	
Location			0.001
oral cavity	246 (60.4%)	30 (28.8%)	
oropharynx	23 (5.7%)	45 (43.3%)	
hypopharynx	10 (2.5%)	6 (5.8%)	
larynx	128 (31.4%)	23 (22.1%)	
History of smoking			0.426
Yes	321 (78.9%)	78 (75%)	
No	86 (21.1%)	26 (25%)	
History of alcohol consumption			0.354
Yes	264 (64.9%)	73 (70.2%)	
No	143 (35.1%)	31 (29.8%)	
Lymphatic metastasis			0.046
Yes	220 (54.1%)	68 (65.4%)	
No	187 (45.9%)	36 (34.6%)	

## Data Availability

The data collected from public databases in this study can be accessed using the accession numbers provided in Section 2. The organized patient data and sequencing data are available upon reasonable request to the corresponding author by email.

## Supplementary Materials

The following supporting information can be downloaded at: https://www.mdpi.com/article/10.3390/cancers18152420/s1. Figure S1: Cell type composition and marker annotation; Figure S2: Identification of metastasis-associated epithelial subgroups in HPV− HNSCC; Figure S3: Functional enrichment analysis of HPV16 early gene-transfected CAL27 cells; Table S1: Clinicopathological characteristics and p16 status of patients with HNSCC; Table S2: Univariate and multivariate logistic regression analyses for lymph node metastasis; Table S3: Overlap analysis between shSERPINB3-upregulated genes and the HPV+ C1 cluster signature; Table S4: Sequences and lentiviral vectors of HPV16 early genes.

## Abbreviations

The following abbreviations are used in this manuscript:

EMT	Epithelial–Mesenchymal Transition
CSCs	Cancer stem cells
HNSCC	Head and Neck squamous cell carcinoma
HPV	Human Papillomavirus
LNM	Lymph node metastasis
SERPINB3	Serpin Family B Member 3
scRNA-seq	Single-Cell RNA Sequencing
PTs	Primary Tumors
MTs	Metastatic Tumors
