# Supplementary material for: HPV16 E7-Associated SERPINB3 Suppression and MYC-Related Epithelial Plasticity in Head and Neck Squamous Cell Carcinoma
Source: Cancers (Basel). 2026 Jul 27;18(15):2420. doi: 10.3390/cancers18152420 (PMC13465601; doi:10.3390/cancers18152420)
Supplement: Supplementary file 1 [file cancers-18-02420-s001.zip › Supplementary Figures.pdf]

Figure S1: Cell type composition and marker annotation

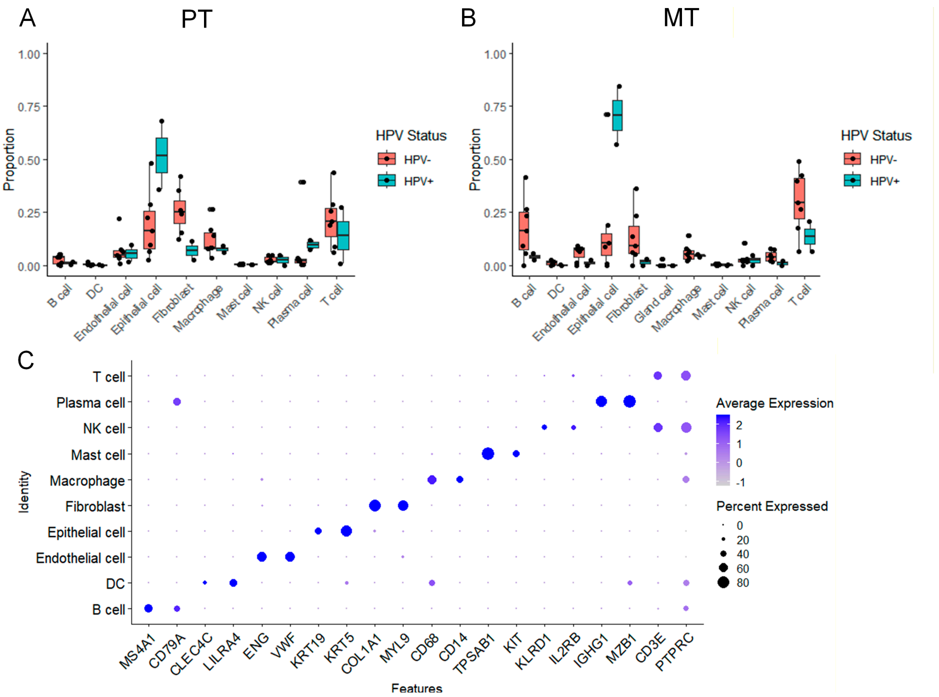

Figure S1. A, B: Box plots comparing the proportion of different cell types in PT (Primary Tumor) and MT (Metastatic Tumor) samples, grouped by HPV (Human Papillomavirus) infection status. C, D. Dot plot showing the expression of canonical marker genes used for cell type annotation.

Figure S2: Identification of metastasis-associated epithelial subgroups in HPV- HNSCC

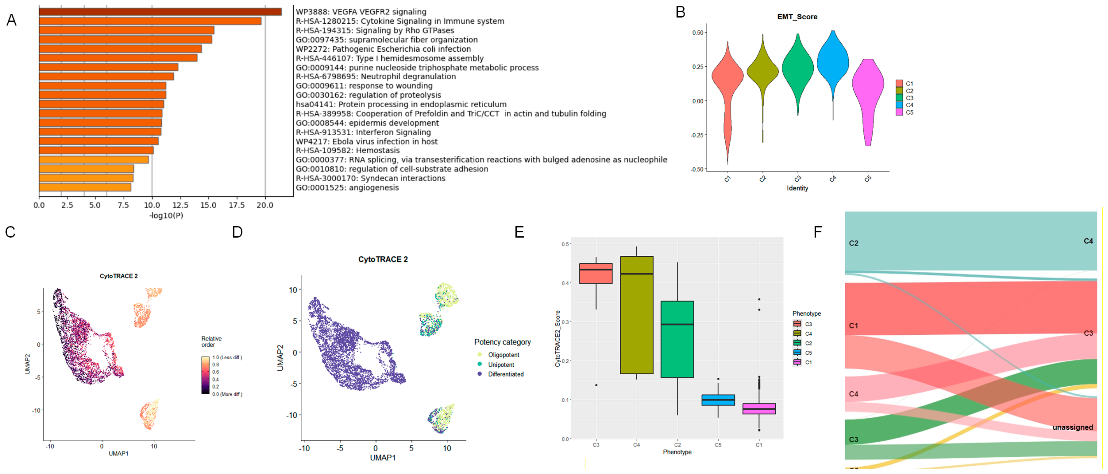

Figure S2. Identification of metastasis-associated epithelial subgroups in HPV- HNSCC lymph node metastases.

A, Functional enrichment analysis of marker genes in the C3 subpopulation. B, Violin plot showing epithelial-mesenchymal transition (EMT) scores across five malignant epithelial subgroups. C, UMAP visualization of CytoTRACE2 relative differentiation order in HPV- metastatic malignant epithelial cells. D, UMAP visualization of CytoTRACE2-predicted potency categories, including oligopotent, unipotent, and differentiated states. E, Box plot comparing CytoTRACE2

relative scores across five malignant epithelial subgroups. F, Sankey diagram showing the correspondence between malignant epithelial subgroups in HPV- primary tumors (PTs) and metastatic tumors (MTs).**

Figure S3: Functional enrichment analysis of HPV16 early gene-transfected CAL27 cells

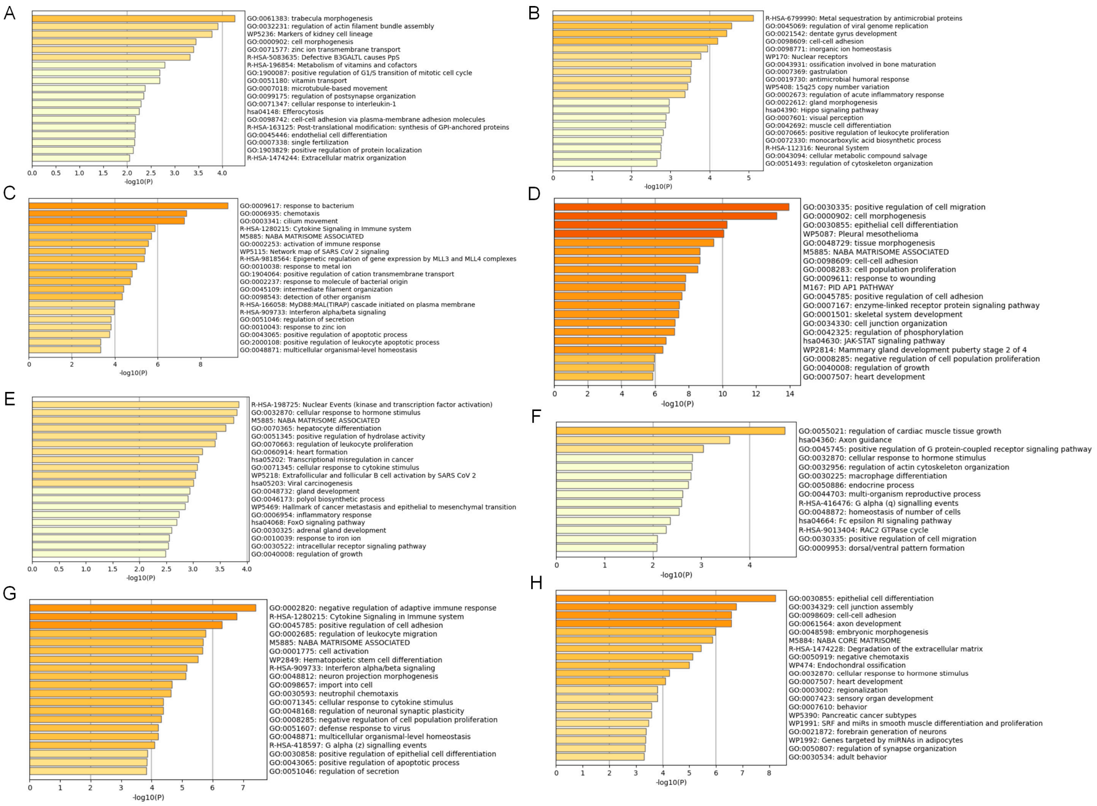

Figure S3. Functional enrichment analysis of HPV16 early gene-transfected CAL27 cells using Metascape. Bar graphs display the top 20 enriched terms (Gene Ontology biological processes and KEGG pathways) for upregulated (left panels) and downregulated (right panels) genes in: A, B: HPV16 E5-transfected cells; C, D: HPV16 E6-transfected cells; E, F: HPV16 E6*-transfected cells; G, H: HPV16 E7-transfected cells. Terms were ranked by statistical significance (-log10-transformed p-values) and filtered ($p < 0.05$). Color intensity in bars reflects the magnitude of significance (darker shades: lower p-values). Redundant terms were removed using Metascape's clustering algorithm.
